# Urban Household Energy Use: Analyzing Correlates of Charcoal and Firewood Consumption in Kampala City, Uganda

**DOI:** 10.1155/2021/5904201

**Published:** 2021-04-08

**Authors:** Abel Nzabona, Richard Tuyiragize, John Bosco Asiimwe, Christian Kakuba, Peter Kisaakye

**Affiliations:** ^1^Makerere University, Department of Population Studies, Kampala, Uganda; ^2^Makerere University, Department of Planning and Applied Statistics, Kampala, Uganda

## Abstract

Charcoal and firewood contribute to greenhouse gas emissions in rural and urban areas. Although there is information about energy types used for cooking in Kampala urban environment, less is known about the correlates of charcoal and firewood consumption. This study investigated the predictors of charcoal and firewood use for cooking using the 2014 Uganda Census data set. Analysis was conducted on 41,250 households in Kampala City. Multinomial logistic regression model was fitted to predict charcoal and firewood use. Findings indicate that older household heads were more likely to use firewood than their younger counterparts. Charcoal and firewood were more likely to be used in households whose household heads were females, married and formerly married, and lived in dwelling units with two and more rooms. Conversely, chances of using charcoal and firewood decreased with the level of education, living in detached house and flat, and residing in shelter with cement screed or tile/concrete. The findings have several implications including long-term planning for improving formal education conditions, strengthening female empowerment, and upgrading dwelling conditions of the households in Kampala City.

## 1. Introduction

Charcoal and firewood dominate the sources of energy used for cooking in developing countries [1]. Over 2 billion people in developing countries are reported to rely on traditional biomass fuels including wood, agricultural residues, and dung for their daily energy needs [2]. Firewood and charcoal are the leading sources of energy for cooking in most of Uganda's households. In 2014, firewood and charcoal contributed 71.2% and 22.7%, respectively, to the sources of energy for cooking in Uganda [3]. Although the use of woodfuel (firewood and charcoal) in urban areas may be less prevalent in comparison with rural areas, the level is quite high even in Kampala City, the nation's capital and largest commercial center.

Energy demand and use have implications on various aspects of the natural and social environment [4–6]. Affordable and reliable energy is vital for meeting basic human needs and can be a corner-stone of development. Having access to modern energy sources such as electricity or liquefied petroleum gas (LPG) can impact human wellbeing by reducing health and safety risks often associated with traditional energy use [7]. Modern energy sources can also translate into decreasing time budget constraints on household members, particularly women and children who usually take substantial time and effort in cooking and collecting firewood [8]. When households move up along the energy ladder and use less of woodfuel, emissions of greenhouse gases tend to decrease [9].

Broad categories of energy have been suggested. A distinction is made between traditional fuels, transition fuels, and advanced fuels [2]. Animal waste, agricultural waste, and firewood are classified as traditional fuels, while charcoal, kerosene, and coal are categorized as transition fuels. The advanced fuels are biofuels, LPG, and electricity. Kowsari and Zerriffi (2011) present a three-dimensional energy profile comprising of traditional fuels (crop residue, woodfuel), transitional fuels (charcoal, kerosene), and modern fuels (LPG, electricity).

Substantial effort has been made towards understanding levels and patterns of woodfuel dynamics [10–12]. Woodfuel consumption levels are quite high [10–13] with consequences that pose risks to the sustainability of the resource. Interest is rising towards deeper reflection on factors with which woodfuel is associated [13–16]. Studies indicate that woodfuel is associated with age [17], education [14], and household location [18]. In the latter study, residence ownership, dwelling category, household income, and education level of household head were other factors found to be important in influencing the choice of fuel.

High level combustion of biomass including charcoal and firewood is a matter of concern for environmentalists considering that combustion is one of the major sources of greenhouse gases [19]. Effects of smoke arising from burning woodfuel are one of the predisposing factors to acute respiratory infections (ARI) in young children [20]. The reliance on biomass for cooking and heating purposes exposes many women and young children in developing countries to high levels of indoor air pollution. To manage these challenges, United Nations has continued to advocate for affordable and clean energy. Goal 7 of the Sustainable Development Goals (SDGs) aims to ensure access to affordable, reliable, sustainable, and modern energy [21].

Trends indicate higher prevalence of charcoal and firewood in rural areas in comparison with Uganda's urban areas. Although the use of charcoal and firewood happens in urban environment, the prevalence in Kampala City is substantially high in spite of rising electricity coverage [3]. There is some evidence of links between household woodfuel consumption and energy insecurity [22], but less is known about the correlates of charcoal and firewood use [23]. Evidence is particularly limited regarding the factors associated with woodfuel use for cooking in urban households. This paper fills the gap by analyzing predictors of charcoal and firewood use in Kampala City. The main objective of the study was to examine the correlates of urban household use of traditional energy (charcoal and firewood) for cooking in Kampala City, Uganda.

## 2. Theoretical & Conceptual Framework

### 2.1. The Energy Ladder Model

**Figure 1:**
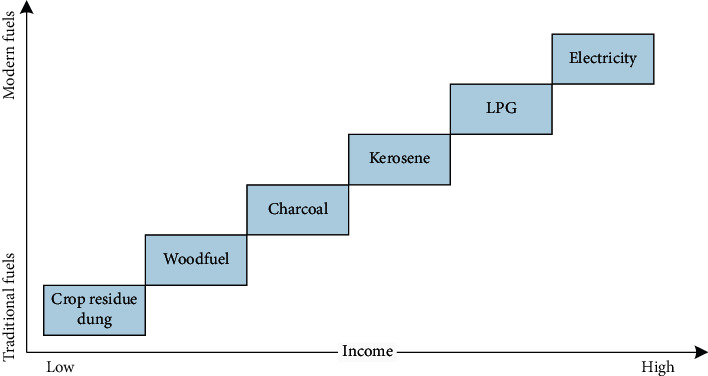
The energy ladder (adapted from: Kowsari & Zerrifi (2011)). Three-dimensional energy profile: a conceptual framework for assessing household energy use (energy policy: Elsevier).

The paper was informed by previous work on the energy ladder model and the hypothesized determinants of household energy choice [7]. The energy ladder model, which was developed based on the correlation between income and uptake of non-solid sources of energy such as electricity, describes a pattern of fuel substitution as a household's economic situation changes [24]. The model ranks fuels based on efficiency, cleanliness, and convenience of storage and usage [7]. Consequently broad categories are suggested, namely, traditional fuels (animal waste, agricultural waste, and firewood), transition fuels (charcoal, kerosene, and coal), and advanced fuels (biofuels, LPG, and electricity) [2]. Traditional fuels are at the bottom of the ladder, while the advanced fuels are at the apex (Figure 1).

The model presupposes that households switch from traditional energy systems at the bottom of the ladder to modern ones higher up the ladder. The shift depends on factors such as household income, fuel and equipment costs, availability and accessibility of fuels, reliability of modern fuel distribution, and relative fuel prices [25]. The model suggests further that as families gain improved socioeconomic status, they abandon first-stage technologies that are inefficient, less costly, and more polluting. Rather, the pattern gradually shifts towards second-phase transition fuels and, ultimately, to the higher-order fuels (LPG and electricity) in the third phase. While higher-order fuels may be costly, they are considered more efficient and may require less labor and produce less pollution per unit of fuel [25].

### 2.2. Factors Determining Household Energy Choice

The conceptual framework used in the paper is informed by previous work on factors determining household energy choice (Kowsari & Zerrifi, 2011). The authors posit that household energy choice depends on household decisions based on a complex interaction between endogenous (household) factors and exogenous factors (external factors). The endogenous factors are economic (such as household/personal income, remittances, economic activity, and remittances), sociodemographic (including age, sex, marital status, and education), and behavioral (such as food taste preferences, practices, and lifestyles). The exogenous factors comprise physical environment (location and type of dwelling unit), energy policies and supply (energy affordability, availability, reliability, tariffs, and subsidies), and energy device characteristics (device conversion efficiency, payment method, and complexity of operation). These factors are deemed to be closely interrelated and tend to operate in conjunction rather than in isolation.

**Figure 2:**
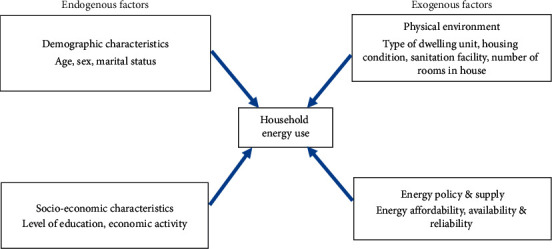
Conceptual framework of factors determining household energy choice (adapted from: Kowsari & Zerrifi (2011)). Three-dimensional energy profile: a conceptual framework for assessing household energy use (energy policy: Elsevier).

In our study, the endogenous demographic factors operationalized are age, sex, and marital status, while the endogenous socioeconomic factors analyzed are education and economic activity (Figure 2). Type of dwelling unit, housing condition, number of rooms in house, and sanitation facility are the exogenous physical factors, while energy affordability, availability, and reliability are the exogenous energy policy/supply factors. We hypothesize that the endogenous and exogenous factors influence the choice of urban household source of energy for cooking. Thus, this study was set out to test three hypotheses: first, charcoal is less likely to be used in households headed by persons of higher levels of education compared with their counterparts with no formal education. Second, firewood is more likely to be used in households headed by females than those headed by males, and last, charcoal is less likely to be used in households living in tenements (one-roomed shelters) than those residing in detached houses.

## 3. Materials and Methods

### 3.1. Source of Data and Sample Size

This paper uses secondary data collected in the 2014 Uganda National Population and Housing Census whose census reference night was set as 27 August 2014. Data were collected from persons who spent the census night in each of the country's households. A household questionnaire was used to collect information on population, socioeconomic, housing, and household-based agricultural characteristics. This was obtained from 112 districts (including Kampala City) that constituted the country. Kampala City was selected for our study being the largest metropolis in the country and a major urban consumer of woodfuel. Household data for Kampala City (41,525 households) were consequently extracted from the main national dataset for this study.

### 3.2. Measurement Variables

Source of energy for cooking was the outcome variable in our study. The census asked questions about the type of energy used for household lighting and cooking. The question that sought information on cooking was: “What source of energy does this household mainly use for cooking?”. The sources of energy captured during the 2014 census were national grid electricity, solar electricity, generator electricity, thermal plant electricity, gas, kerosene, charcoal, firewood, cow dung, grass, and others. Owing to extremely low frequencies on some sources, re-coding of energy source was done during analysis to yield four major categories, namely, charcoal, firewood, electricity, and ‘others.' The latter category includes gas, kerosene, grass, reeds, and cow dung. Energy for cooking, the dependent variable, was analyzed with several independent variables. These were age, sex, marital status, level of education, economic activity, type of dwelling unit, number of rooms in dwelling unit, shelter floor material, and toilet facility.

### 3.3. Data Analysis

Frequency distributions of energy types were compiled to determine levels of energy types used for cooking among households in Kampala City. Frequencies by background characteristics were also compiled. Multinomial logistic regression model was fitted to isolate the predictors of household charcoal and firewood use. This was done considering that the dependent variable had several response categories, namely, charcoal, firewood, electricity, and others. Relative Risk Ratios (RRR) were computed to analyze the chances of using charcoal and firewood in comparison with electricity, which was set as the base outcome. A correlation test was performed, and results point to no or minimal correlation for variables included in the model.

### 3.4. Ethical Issues

As indicated earlier, our study used secondary data collected in the national census. The Uganda Bureau of Statistics (UBOS), which is the National Statistical Office (NSO) that conducts national censuses, makes public data collected in censuses and surveys. National institutions of higher learning are particularly encouraged to use these data for research and other academic purposes. We were, therefore, able to freely access the data from the NSO for our analysis.

## 4. Results

### 4.1. Energy Choices

Table 1 indicates that over three-quarters (77.8%) of the households in Kampala City mainly used charcoal for cooking. About three percent (2.6%) used firewood, bringing the overall usage of woodfuel (charcoal and firewood) among households to 80.4%. The percentage using electricity and other fuels was 8.2 and 11.5, respectively.

### 4.2. Background Characteristics of Household Heads

Results in Table 2 indicate that the majority of the respondents were aged 30–34 (42%) followed by those in the 15–29 age bracket (38%), while those aged 45–59 and 60–95+ were 16% and 5%, respectively. Seven in ten were males, while just 3 in 10 were females. About 6 in 10 (60%) were married, while one-quarter (25%) were never married.

Regarding education, nearly half (48%) had secondary education, while just over one-fifth (22%) had primary education. Those with tertiary and higher education were 11% and 16%, respectively. Just over half (53%) were paid employees, while slightly over a quarter (27%) were own-account workers. The percentage of those who were employers and unemployed was 5% and 4%, respectively. Only about a quarter had received remittances in kind and cash, and almost all were not involved in any household-based enterprise (99%). Table 2 shows other distributions by dwelling unit, floor material, number of rooms in dwelling unit, and toilet facility.

### 4.3. Predictors of Urban Household Energy Use

A multinomial logistic regression model was fitted to analyze predictors of energy used for household cooking. The factors that significantly influenced the type of energy were age, sex, marital status, education, type of dwelling unit, number of rooms, and type of toilet facility (Table 3). The predictors are presented at 0.01% significance level and in reference to *electricity*, which is the base outcome.

In comparison with persons aged 15–29, the chances of using firewood were increased for persons aged 45–49 (RRR = 1.99) and 60+ (RRR = 4.13). Being female increased the chances of charcoal and firewood use (RRR = 1.52 and RRR = 1.30, respectively) but reduced the likelihood of using other energy types (RRR = 0.63). Being married and formerly married increased the chances of using charcoal and firewood in comparison with being never married. The likelihood of using charcoal for those who were married and never married was RRR = 1.94 and RRR = 1.79, respectively. However, being married and never married decreased the chances of using other types of energy (RRR = 0.34 and RRR = 0.65, respectively).

In comparison with persons who did not have formal education, the chances of using charcoal reduced with increasing level of education. The chances of those with secondary, tertiary, and degree level of education were RRR = 0.71, RRR = 0.62, and RRR = 0.56, respectively. Similarly, the likelihood of using firewood reduced with education. In comparison with those who did not have formal education, the chances were lower for those with primary, secondary, tertiary, and degree level of education (RRR = 0.97, RRR = 0.37, RRR = 0.29, and RRR = 0.16, respectively). However, education was associated with increased chances of using other energy sources for persons of degree level of education (RRR = 2.25).

Compared to living in tenement, residing in detached/semi-detached house, flat, and other type of dwelling unit reduced the chances of using charcoal (RRR = 0.45, RRR = 0.14, and RRR = 0.19, respectively). Only living in flat reduced the chances of using firewood. The chances of using other types of energy were also reduced by living in detached, flat, and other types of dwelling units. The number of rooms in a dwelling unit also influenced energy use. Living in two rooms and three rooms and above increased the chances of using charcoal (RRR = 1.32 and RRR = 1.52, respectively).

Table 3 further indicates that living in shelter with title or concrete floor reduced the chances of using charcoal and firewood (RRR = 0.69 and RRR = 0.21, respectively) compared with living in shelter with rammed earth floor. Residing in shelter with cement screed and other floor material also reduced the chances of using firewood (RRR = 0.32 for both categories). However, living in shelter with tile or concrete floor increased the chances for using other types of energy. In comparison with shared toilet facility, having a not shared toilet facility reduced the chances of using charcoal (RRR = 0.80) but increased the likelihood of using other types of energy (RRR = 1.23).

## 5. Discussion

The main objective of the study was to examine urban household energy use for cooking in Kampala City, while the specific objective was to analyze the correlates of charcoal and firewood consumption. Findings indicate that the likelihood of firewood use increased with age. The trend/pattern may relate to the tendency for some older persons to maintain the practice of using traditional fuels as relatively younger persons switch to more modern forms of energy. Other studies have shown that older persons restrain to move away from their current practices [2]. Age has similarly been found to be one of the socioeconomic factors that promote popularity and use of woodfuel [17].

As hypothesized, charcoal was less likely to be used in households headed by persons of higher levels of education compared with their counterparts who did not have formal education. It is likely that as education increases, the probability of access to resources improves. This could enable households to afford cleaner energy sources. Households headed by persons of higher education may also have had less time to spend on chores, probably due to engagement in the formal sector; hence, the use of less time-consuming energy sources. Conversely, those with no formal education may have engaged more with charcoal use probably owing to their relative low affordability of cleaner sources. Education has similarly been found to be a strong predictor of cooking fuel choice in India's states of Kerala and Rajasthan [14] and wood consumption in Brazil's Atlantic Forest Area [26].

Female-headed households were more likely to use firewood for cooking (as hypothesized) than their male-headed counterparts. The higher likelihood is perhaps expected considering that, in the Ugandan setting, a large proportion of females are disproportionately under-resourced in comparison with their male counterparts. The disadvantage could have translated into limited household resources for affording cleaner sources of energy. Studies elsewhere have similarly indicated that a large proportion of female-headed households tend to have compromised ability to access modern fuels, resorting to the comparatively less costly woodfuel sources [27]. The higher likelihood of using charcoal and firewood among the currently married and ever married persons could be explicable in terms of differences in domestic roles and responsibilities. For example, while the married could have headed sizable households with greater demand on food preparation, some of the never married persons may, conversely, have headed smaller households where cooking demands were less, and, hence, it was possible to use alternative and more convenient forms of energy such as electricity and gas.

Economic activity influenced energy use with the unemployed having more chances of using woodfuel. Conversely, households in which there was some household enterprise had reduced chances of using woodfuel. Variations in financial status and capacity to afford cleaner energy sources may have explained the disparity. This finding appears to dovetail with the energy ladder hypothesis, which asserts that as income rises, energy choices shift towards less traditional fuels [7]. Income level has been reported to be a major factor influencing household energy utilization and changing behaviors in Western Kenya [28].

Contrary to our hypothesis, charcoal was more likely to be used for cooking in households living in tenements (one-roomed shelters) than those residing in detached houses. It may be the case that those living in tenements were of lower socioeconomic status and, hence, less able to afford cleaner energy sources than those residing in detached houses. Other plausible explanation relates to the link between dwelling unit and house occupancy tenure. In the setting of Uganda, particularly Kampala City, dwelling unit and house occupancy tenure tend to be associated, with those living in detached houses likely to be owner occupiers, while those in tenements likely to be renters. Some renters could be handicapped in the event that the landlord did not make necessary provisions for accessing cleaner sources of energy such as electricity. Studies on relationship between house occupancy tenure and household energy use [29] have indicated that being the owner of a house does not necessarily imply having higher purchasing power but could mean prevalence of freedom of space management in the house. Tenants may have less choice as they are expected to comply with occupancy contracts and regulations which may compromise their leeway in deciding energy options.

In comparison with living in houses with rammed earth floor, living in shelters with cement screed and tile/concrete floor was associated with lower chances of using woodfuel. This finding suggests that the quality of housing influences energy use. Results further show that the likelihood of using charcoal increased with the number of rooms in house. This could be related to space availability for storage and use of charcoal. This dovetails with studies elsewhere, which indicate that house size, measured by the number of rooms, is associated with traditional and transitional energy use [2]. Findings also indicate that households whose toilet facilities were not shared were more likely to use charcoal than those for whom it was shared. Overall, it is probable that the underlying factor for differentials in charcoal use by housing quality, space, and nature of toilet facility is the economic factor.

## 6. Conclusion and Implications

Kampala City households are at the lower end of the energy ladder characterized by charcoal and firewood as the leading sources of energy for cooking. Education influences household energy use with households headed by persons of higher levels of education being more likely to use charcoal than their counterparts with no formal education. The sex of the household head influences energy use with female-headed households being more likely to use firewood than their male-headed counterparts. The type of dwelling unit is a correlate of energy use with households living in tenements more likely to use charcoal than their counterparts residing in detached houses. The findings have several implications including long-term planning for improving formal education conditions, strengthening female empowerment, and upgrading dwelling conditions of the households in Kampala City.

### 6.1. Limitations

The 2014 National Census Dataset used for this study lacked key variables, which would have enriched the study. Consequently, we were unable to analyze wealth index and the effect of the endogenous factors, namely, energy policy and supply, as well as behavioral and cultural characteristics such as food preferences and taste, practices, lifestyles, and cultural diversity. Similarly, we were unable to analyze the role of energy device characteristics such as conversion efficiency, cost, payment method, and complexity of operation.

## Figures and Tables

**Table 1:** Distribution of households by energy mainly used for cooking, Kampala City.

Energy type	Freq	Percent
Charcoal	32,294	77.8
Firewood	1,075	2.6
Electricity	3,397	8.2
Others*∗*	4,759	11.5
**Total**	**41,525**	**100.0**

*∗*Gas, kerosene, grass, cow dung, etc.

**Table 2:** Background characteristics of household heads.

Characteristic	Number	Percent
*Age*		
15–29	15,713	37.8
30–44	17,295	41.7
45–59	6,464	15.6
60+	2,053	4.9

*Sex*		
Male	29,069	70.0
Female	12,456	30.0

*Marital status*		
Never married	10,548	25.4
Married	25,060	60.4
Formerly married	5,917	14.3

*Education*		
No education	1,720	4.1
Primary	9,203	22.2
Secondary	19,767	47.6
Tertiary	4,395	10.6
Degree	6,440	15.5

*Economic activity*		
Employed	35,562	85.6
Unemployed	5,963	14.4

*Dwelling unit*		
Tenement/*Muzigo*^1^	23,560	56.7
Detached^2^	15,091	36.3
Flat	1,546	3.7
Others	1,328	3.2

*Floor material*		
Rammed earth	2,003	4.8
Cement screed	34,615	83.4
Tiles/Concrete	4,602	11.1
Others	305	0.7

*Rooms in dwelling unit*		
1 Room	28,104	67.7
2 Rooms	8,131	19.6
3+ Rooms	5,290	12.7

*Toilet facility*		
Shared	27,620	66.8
Not shared	13,726	33.2

Total	**41,525**	**100.0**

^1^ *One-roomed dwelling unit in larger structure*. ^2^*Includes semidetached house & rooms in main house*.

**Table 3:** Multinomial regression analysis of predictors of urban household energy use.

Characteristic	Charcoal		Firewood		Other^*1*^	
	RRR	[95% CI]	RRR	[95% CI]	RRR	[95% CI]
*Electricity (Base)*						
*Age*						
15–29#	1.00	.	1.00	.	1.00	.
30–44	1.08	0.98–1.12	1.21^*∗*^	0.98–1.49	0.86^*∗∗*^	0.77–0.97
45–59	1.02	0.90–1.16	1.99^*∗∗∗*^	1.57–2.53	0.85^*∗∗*^	0.72–1.00
60+	1.05	0.87–1.28	4.13^*∗∗∗*^	3.06–5.56	0.79^*∗*^	0.61–1.02

*Sex*						
Male#	1.00	.	1.00	.	1.00	.
Female	1.52^*∗∗∗*^	1.38–1.67	1.30^*∗∗∗*^	1.08–1.57	0.63^*∗∗∗*^	0.56–0.71

*Marital status*						
Never married#	1.00	.	1.00	.	1.00	.
Married	1.94^*∗∗∗*^	1.76–2.13	1.49^*∗∗∗*^	1.20–1.85	0.34^*∗∗∗*^	0.31–0.39
Formerly married	1.79^*∗∗∗*^	1.54–2.07	1.71^^*∗∗∗*^^	1.31–2.23	0.65^*∗∗∗*^	0.54–0.79

*Education*						
No education#	1.00	.	1.00	.	1.00	.
Primary	1.13	0.89–1.42	0.97^*∗∗∗*^	0.70–1.32	1.15	0.84–1.57
Secondary	0.71^*∗∗∗*^	0.57–0.88	0.37^*∗∗∗*^	0.27–0.51	0.92	0.69–1.24
Tertiary	0.62^*∗∗∗*^	0.49–0.79	0.29^*∗∗∗*^	0.20–0.42	1.34^*∗*^	0.98–1.83
Degree	0.56^*∗∗∗*^	0.44–0.70	0.16^*∗∗∗*^	0.11–0.24	2.25^*∗∗∗*^	1.65–3.05

*Economic activity*						
Employed	1.00	.	1.00	.	1.00	.
Unemployed	0.93	0.83–1.03	1.30^*∗∗∗*^	1.07–1.58	1.26^*∗∗∗*^	1.11–1.44

*Dwelling unit*						
Tenement/Muzigo#	1.00	.	1.00	.	1.00	.
Detached^***2***^	0.45^*∗∗∗*^	0.40–0.49	0.83^*∗*^	0.68–1.02	0.48^*∗∗∗*^	0.42–0.54
Flat	0.14^*∗∗∗*^	0.12–0.17	0.14^*∗∗∗*^	0.08–0.25	0.63^*∗∗∗*^	0.52–0.77
Others	0.19^*∗∗∗*^	0.16–0.22	0.84	0.62–1.12	0.28^*∗∗∗*^	0.22–0.35

*Rooms in dwelling unit*						
1 room#	1.00	.	1.00	.	1.00	.
2 rooms	1.32^*∗∗∗*^	1.19–1.46	1.57^*∗∗*^	1.29–1.91	0.78^*∗∗*^	0.68–0.89
3+ rooms	1.52^*∗∗∗*^	1.33–1.73	1.99^*∗∗*^	1.59–2.50	0.81^*∗∗*^	0.69–0.96

*Floor material*						
Rammed earth#	1.00	.	1.00	.	1.00	.
Cement screed	0.99	0.82–1.19	0.32^*∗∗∗*^	0.25–0.41	1.07	0.83–1.38
Tiles/concrete	0.69^*∗∗∗*^	0.56–0.85	0.21^*∗∗∗*^	0.15–0.29	2.28^*∗∗∗*^	1.73–2.99
Others	0.68^*∗*^	0.45–1.02	0.32^*∗∗∗*^	0.16–0.65	1.21	0.72–2.04

*Toilet facility*						1.00
Shared	1.00	.	1.00	.	1.23^*∗∗∗*^	
Not shared	0.80^*∗∗∗*^	0.72–0.89	1.27^*∗∗*^	1.04–1.55	1.00	

^*∗∗∗*^Significant at 1% level. ^*∗∗*^Significant at 5% level. ^*∗∗∗*^Significant at 10% level # Reference category CI = Confidence Interval. ^**1**^Gas, kerosene, grass, cow dung, etc. ^**2**^Semidetached house and rooms in main house.

## Data Availability

The data used to support the findings of this study are available from the corresponding author upon request.
